# Dr. Maxwell's Remarks on Sea-Sickness

**Published:** 1826-07

**Authors:** 


### SEA-SICKNESS.*

In No. 2 of our *junior* co-temporary of the North are some observations on sea-sickness, by Dr. Maxwell, which we shall notice here.

Dr. M. observes, what is well known, that this unpleasant affection is always preceded by depressed energy of the system, marked by blanching of the face and hands—lightness of head, &c. He then proceeds to remark, that those most subject to sea-sickness are generally people of strong and healthy constitutions, whilst persons of irritable habits, take phthisical patients for instance, are far less liable to be attacked. With regard to the *modus* operandi of the rolling of the vessel, Dr. M. considers it to be the same as that of "any other emetic," which to be sure is throwing no great light on the matter. Dr. Johnson has ascribed sea-sickness, in a great measure, to the impression produced on the optic nerve, and transmitted to the brain, by the continued motion of the vessel, sails, shrouds, &c. and remarked, that by keeping the eyes closed, this disagreeable nausea is very frequently prevented. This view of the subject was well illustrated by Mr. Bell, in his late lectures at the College of Surgeons. A country gentleman, said he, just arrived in town, will be walking along the Strand, where "the great tide of human existence" sets in. Before, however, he has reached Temple-Bar, the optic nerve, fatigued by the dizzying impression made on it by the continual passing and repassing of objects, will transmit this impression to the brain in such a degree, that the gentleman will reel and actually fall in a state of syncope. So it is in looking from great heights, or fixing the eyes steadily on an object for a length of time; the exertion of the nerve of vision, and the consequent lassitude are communicated to the brain, producing sickness or faintness, as the case may be. As for the remedies, Dr. M. recommends stimulants, such as brandy—walking about, and the horizontal posture—the indication is, to counteract the collapse which is the fore-runner of the attack.

---

* Edinburgh Journal of Medical Science, No. II.

